# Alternative crossing technique for iliaco-femoro-popliteal CTOs with a catheter only

**DOI:** 10.1186/s42155-019-0065-1

**Published:** 2019-07-18

**Authors:** Marc Cunier, Arash Najafi, Gabriel T. Sheikh, Christoph A. Binkert

**Affiliations:** Department of Radiology and Nuclear medicine, Canton Hospital Winterthur, 8401 Winterthur, Switzerland

**Keywords:** Recanalization, Chronic total occlusion, Peripheral arterial intervention, Catheter first, Lower extremity

## Abstract

**Background:**

The standard approach for crossing peripheral CTOs is to use a combination of hydrophilic guidewires and catheters. The path is either intraluminally or in most cases at least partially subintimal. This standard approach with a guidewire-tip as leading point (“wire first”) to cross CTOs has a success rate of about 80%. We hypothesize that a “catheter first” approach, using the catheter alone for the entire recanalization till re-entering the vessel is less traumatic and might lead to a longer intraluminal recanalization due to a softer leading point. Based on this assumption we analyzed the success and duration of this approach with a gradual step-up approach from catheter tip to guidewire front-end to guidewire back-end. To the best of our knowledge, no studies measuring the time of recanalization of lower extremity CTOs using conventional devices were published yet.

**Results:**

Data of 46 consecutive chronic total iliaco-femoro-popliteal occlusions in 43 symptomatic patients treated by percutaneous transluminal angioplasty were collected prospectively between May 1st 2014 and June 30th 2016 and evaluated retrospectively. Chronic occlusion was defined as clinical symptoms or imaging features lasting more than 1 month.

Patient age and gender, diabetes status, localization of occlusion, occlusion length, duration of symptoms, severity of vessel calcification, and recanalization time were assessed.

Technical success was defined as placement of a catheter beyond the distal end of the lesion into the true lumen, confirmed by contrast injection.

All 46 CTOs were successfully recanalized. In 22 cases (47.8%) recanalization was successful with the catheter tip only without the use of a guide wire. In 17 cases (36.9%) the guide wire was used in addition to the catheter. Localization of occlusion did not have an effect on the recanalization technique (*p* = 0.915). The mean rank for length of occlusion was not significant for different recanalization techniques (*p* = 0.095). The success rate for the catheter only approach was lower for higher grades of calcification (*p* = 0.008). There was no correlation between time of recanalization and length of occlusion (Pearson’s *r* = 0.004; adjusted R square = − 0.024; *p* = 0.980), diabetes (*p* = 1.000), sex (*p* = 0.244), or grade of calcification (*p* = 0.621). Recanalization time is significantly right-skewed with most recanalizations being successful under 30 min.

**Conclusion:**

This “catheter first” approach is somewhat contradictory to the prevailing dogma of “wire first”. The concept to use the catheter to start a recanalization is well known, but to perform the entire recanalization including the re-entry seems possible and potentially less traumatic, likely leading to a longer intraluminal course. Our data shows that recanalization of occluded lower extremity arteries between the aortic bifurcation and the popliteal artery can be achieved in the majority of cases (84.7%) solely by using an angled angiographic catheter +/− glide wire.

We suggest a “5 min – 15 min – 30 min” rule on how long to attempt each recanalization technique. More precisely, we suggest trying 5 min with the catheter alone, then 10 min with the soft end of the guidewire and then switching to the stiffer back-end of the guidewire for another 15 min.

## Background

Endovascular recanalization has become the standard treatment in chronic total occlusions (CTOs) (Norgren et al. 2007) due to its minimal-invasiveness and lower periprocedural risk, as outlined in the TASC classification. There are various definitions for chronic total occlusions (CTOs). The most widely accepted definition refers to the coronary vessels in which a CTO is defined as a lesion with no antegrade flow that is present for an (estimated) duration of more than 3 months, meaning 100% blockage of the vessel (Kirvaitis et al. 2007). This is in contrast to peripheral CTOs where most authors define lesions as chronic if symptoms have been present for at least 30 days (Laird et al. 2014; Bosiers et al. 2014).

The primary and most common approach for crossing CTOs is to use a combination of hydrophilic guidewires and catheters to cross the occlusion either intraluminally or as in most cases (80%) at least partially subintimal (London et al. 2011; Chen et al. 2011; Molloy et al. 2003). To intentionally recanalize a CTO of the superficial femoral artery subintimally, the catheter tip can be used to enter the subintimal space and in order to stay extraluminally, a guide wire in large loop configuration is used to proceed down through the natural dissection plane (Reekers and Bolia 1998). Success rates for recanalization attempts using this standard approach with a guidewire-tip as leading point (“wire first”) to cross peripheral CTOs have reported success rates of about 80% (Carnevale et al. 2004; Löfberg et al., n.d.). For subintimal angioplasty of femoro-popliteal occlusions the same technical success rate of 80% have been reported (Reekers and Bolia 1998; London et al. 2011). Main reasons of failure are not passing the occlusion and/or not re-entering the true lumen after subintimal recanalization.

There are several hypothetical advantages to remaining in the vessel lumen, mainly reduced risk of perforation and flow limiting dissection as well as conservation of collateral vessels (Laird et al. 2014).

We hypothesize that a “catheter first” approach for the entire length of the recanalization is less traumatic and might also lead to longer intraluminal recanalization due to a softer leading point. Based on this assumption we analyzed the success and time consumption of this approach with a gradual step-up to guidewire front- and back-end. It is important to note the difference between this approach and the above mentioned technique, where the catheter tip is used to enter the subintimal space. To the best of our knowledge, no studies measuring the time of recanalization of lower extremity CTOs using conventional devices were published.

## Materials and methods

Data of 46 chronic total iliac, femoral, and popliteal occlusions in 43 symptomatic patients treated by percutaneous transluminal angioplasty was collected prospectively between May 1st 2014 and June 30th 2016 and evaluated retrospectively. Chronic occlusion was defined as clinical symptoms or imaging features lasting more than 1 month. Written informed consent for the procedure and data analysis were obtained from all patients.

### Catheter first approach

All cases started with the blunt / atraumatic tip of an angled angiographic catheter (KMP, Cook Medical, Bloomington IN, USA) as the leading point to cross the entire length of the occlusion including the access to the true lumen using the guide wire only as catheter support within the shaft of the catheter. If this technique failed, the tip of an angled guidewire (Glidewire, Terumo Medical Corporation, Somerset NJ, USA) was used (equaling the standard “wire first” approach). If recanalization was not possible, the stiffer back end of the glidewire was utilized. If these attempts were unsuccessful, different wires, retrograde recanalization and/or re-entry devices were deployed.

### Data collection

**Fig. 1:**
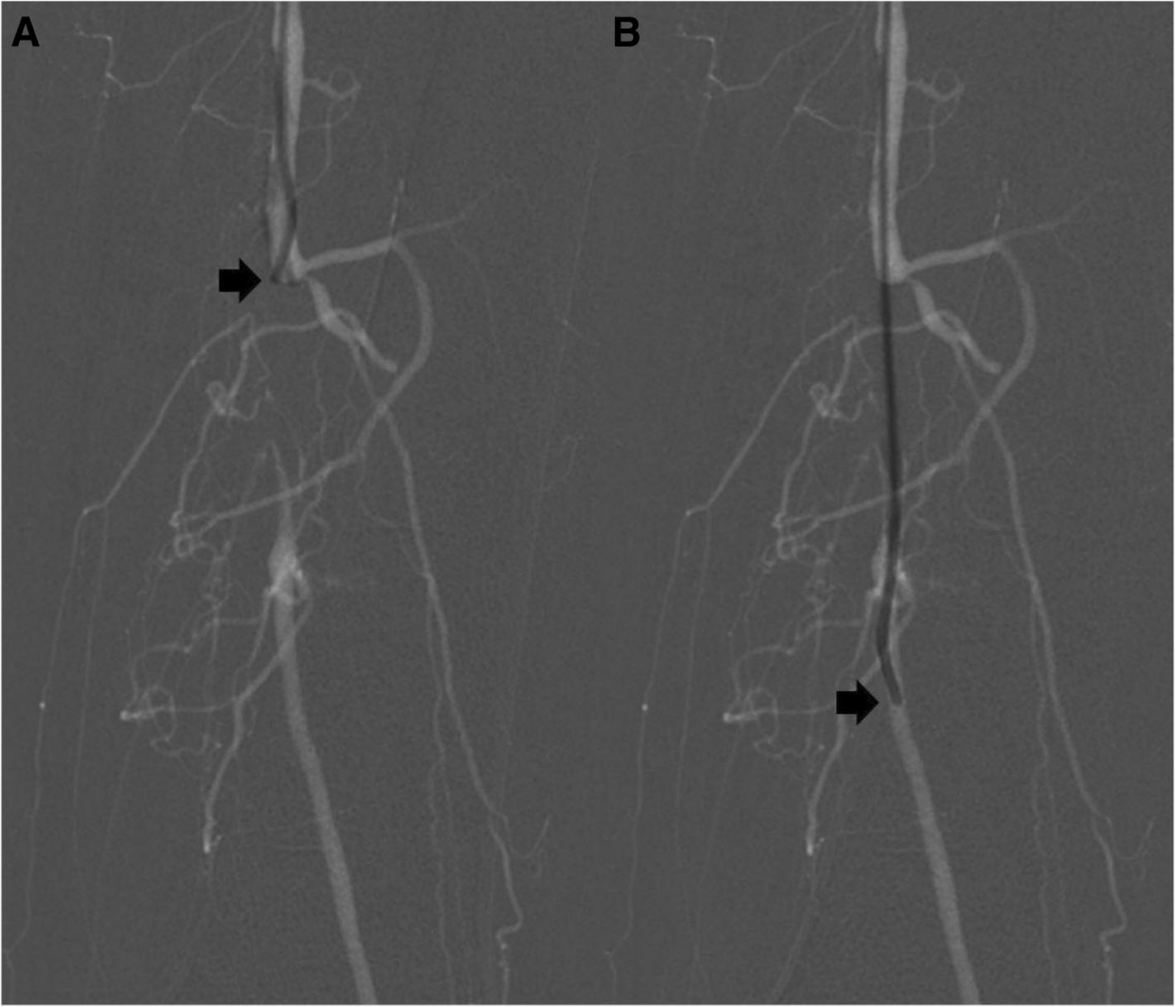
Recanalization of a femoral occlusion with the tip of a KMP catheter only. **a** Roadmap image prior and **b** after crossing of a chronic total occlusion of the left femoral artery with a “catheter first” approach without the use of a guide wire showing the position of the catheter tip (black arrows)

Collected data included patient age and gender, diabetes status, localization of occlusion (iliac artery, superficial femoral artery, or popliteal artery), occlusion length, duration of symptoms, severity of vessel calcification (none, mild, and severe by the interventional radiologists using simplified Stoner criteria (Stoner et al. 2016)), and recanalization time (measured by the time difference between the roadmap image before the recanalization and test injection in the artery distal to the occlusion, Fig. 1). Technical success was defined as placement of a guide wire beyond the distal end of the lesion into the true lumen, confirmed by contrast injection.

### Statistical analysis

For quantitative variables, number of observations (N), minimum (Min), maximum (Max), mean, standard deviation (SD), median, first quartile (Q1), third quartile (Q3), and interquartile range (IQR) are given. For categorical variables, absolute (N) and relative frequencies (%) are shown. Patients treated with different recanalization techniques were compared with a Fisher’s exact test for nominal data and a Kruskall-Wallis test for ordinal or interval scaled variables. When *p*-value was ≤0.05, post-hoc tests were performed, i.e. column proportions were compared using a z-test and mean ranks were compared using a Mann-Whitney U test. None of the tests were controlled for multiple testing. All analyses were done using SPSS (IBM SPSS Statistics Version, n.d.) Version 25.

## Results

Patient demographics and baseline data are shown in Table 1.

**Table 1:** Demographics and baseline data of included patients (*N* = 43)

Variable	Patients (*N* = 43)
Male sex (%)	22 (51.2%)
Age, mean years (sd)	71.2 (±14.6)
Diabetes (%)	11 (25.6%)

All 46 CTOs were successfully recanalized. In 22 cases (47.8%) recanalization was successful with only the catheter first approach. In 17 cases (36.9%) the glidewire was used in addition to the catheter. A more detailed listing of the success-rates of the recanalization techniques is presented in Table 2.

**Table 2:** Success of recanalization technique

Recanalization technique	N (%)
KMP only	22 (47.8)
KMP + soft end GW	11 (23.9)
KMP + back end GW	06 (13)
Other	07 (15.3)
Total	46 (100)

Recanalization techniques did not differ between the localization of occlusion (*p* = 0.915, Table 3).

**Table 3:** Contingency table for localization of occlusion and recanalization technique. Results show absolute and relative frequencies

Localization of occlusion	Recanalization technique (%)				Total
	KMP only	KMP + soft end GW	KMP+ back end GW	Other	
Iliacal	4 (50.0)	2 (25.0)	0 (0.0)	2 (25.0)	8 (100.0)
Femoral	10 (38.5)	7 (26.9)	5 (19.2)	4 (15.4)	26 (100.0)
Femoral+ popliteal	5 (62.5)	1 (12.5)	1 (12.5)	1 (12.5)	8 (100.0)
Popliteal	3 (75.0)	1 (25.0)	0 (0.0)	0 (0.0)	4 (100.0)
Total	22 (47.8)	11 (23.9)	6 (13.0)	7 (15.3)	46 (100.0)

The mean rank for length of occlusion was not significant for different recanalization techniques (*p* = 0.095, Table 4).

**Table 4:** Descriptive statistics for length of occlusion (cm) stratified by recanalization technique

Success	N	Missing	Min	Max	Mean	SD	Median	Q_1_	Q_3_	IQR
KMP only	22	0	1.0	25.0	8.1	4.9	8.0	5.8	9.6	4.2
KMP + soft end GW	11	0	3.0	34.0	14.4	10.5	12.0	6.0	24.5	18.5
KMP + back end GW	6	0	1.0	22.0	7.2	7.9	5.2	1.4	11.6	10.2
Other	7	0	5.0	22.0	13.9	6.7	15.0	6.5	21.0	14.5
Total	46	0	1.0	34.0	10.4	7.6	8.0	5.8	13.6	7.8

**Table 5:** Contingency table for grade of calcification and recanalization technique. Results show absolute and relative frequencies

Grade of calcification	Recanalization technique (%)				Total
	KMP only	KMP + soft end GW	KMP + back end GW	Other	
None	8 (72.7)	3 (27.3)	0 (0.0)	0 (0.0)	11 (100.0)
Mild	10 (52.6)	5 (26.4)	2 (10.5)	2 (10.5)	19 (100.0)
Severe	4 (25.0)	3 (18.8)	4 (25.0)	5 (31.2)	16 (100.0)
Total	22 (47.8)	11 (23.9)	6 (13.0)	7 (15.2)	46 (100.0)

Recanalization technique was distributed significantly different between patients with varying grades of calcification (*p* = 0.008) with a lower success rate for the catheter only approach for higher grades of calcification (Table 5). Post-hoc tests indicated that grade of calcification was significantly different between patients treated with catheter only and catheter + back end GW (*p* = 0.024) and catheter only and other recanalization techniques (*p* = 0.11). There was no significant difference in grade of calcification between the soft and the stiff end GW (*p* = 0.122).

Recanalization time is significantly right-skewed with two extreme outliners that required more than 75 min (or twice the previous maximum), both times in cases with the use of a re-entry devices. Excluding these extreme outliners, the rest of the data (*N* = 43) is still significantly right-skewed (skewness = 2.631, std. deviation = 0.361). This indicates that the catheter only approach was fast if successful. There was no difference in recanalization time related to diabetes (*p* = 1.000), sex (*p* = 0.244), or grade of calcification (*p* = 0.621). There was no correlation between time of recanalization and length of occlusion (Pearson’s r = 0.004; adjusted R square = − 0.024; *p* = 0.980).

The actual median recanalization time was 2:04 min. For catheter only, 5:14 min. For catheter with soft tip GW and 4:34 min. For catheter with stiff tip GW (see Table 6 below).

**Table 6:** Descriptive statistics for time of recanalization (min:sec) stratified by technique

Success	N	Min	Max	Mean	SD	Median	Q_1_	Q_3_	80th p.	IQR
KMP only	22	0:25	09:39	2:58	2:22	2:04	1:22	3:56	5:44	2:34
KMP + soft end GW	11	1:37	21:41	7:23	6:21	5:14	1:53	11:51	12:49	9:58
KMP + back end GW	6	1:36	37:27	10:39	13:36	4:34	3:12	18:22	27:16	15:10
Other	4	3:27	30:00	13:22	12:28	10:01	3:37	26:29	N/A	22:52
Total	43	0:25	37:27	6:08	7:41	3:20	1:37	6:48	9:32	5:11

**Fig. 2:**
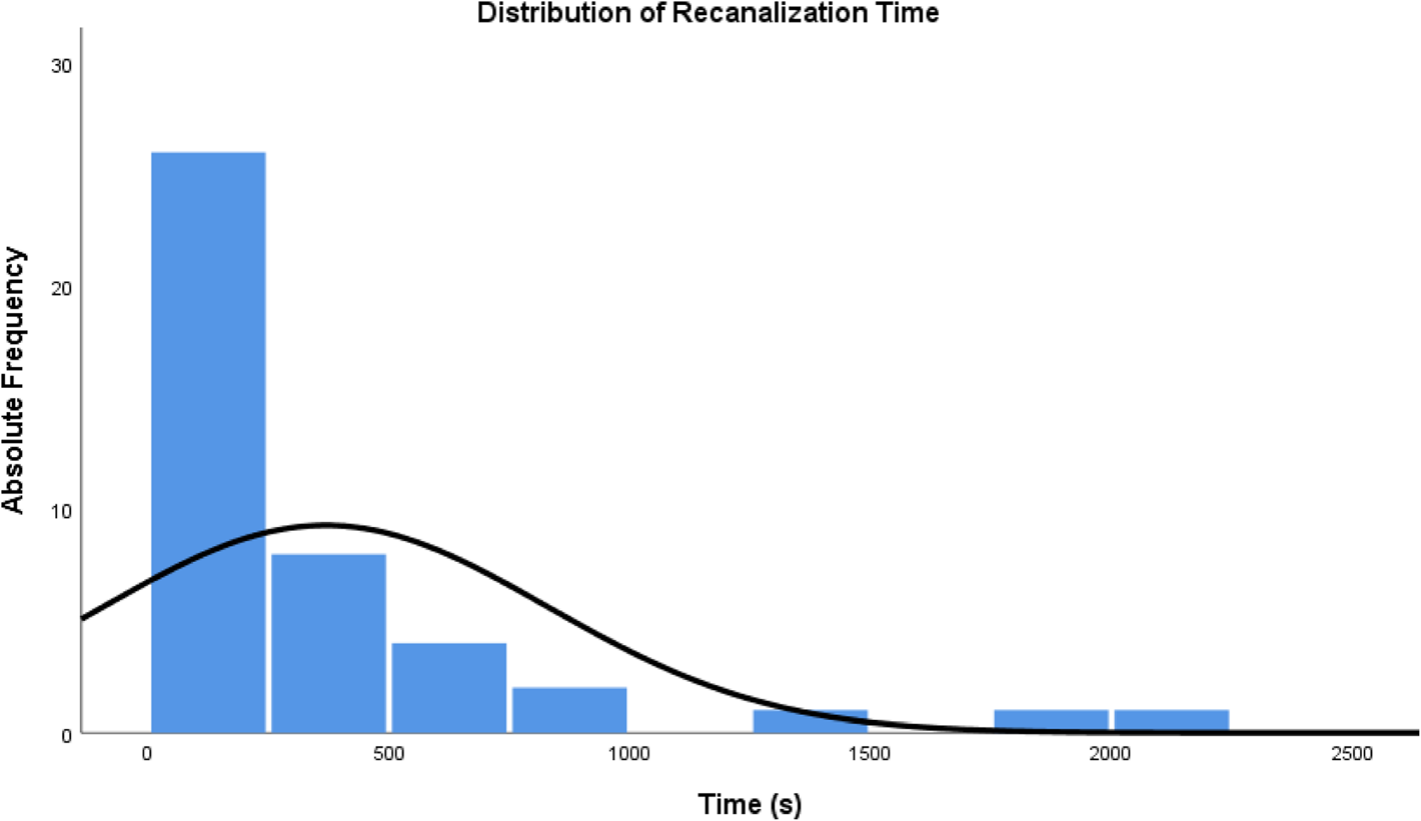
Distribution of recanalization time

Distribution of recanalization time is shown in Fig. 2.

Only in 1 CTO this approach caused a flow limiting dissection in the starting point of recanalization beyond the extent of the target lesion, which was easily treated with a stent reaching 2 cm proximal of the former occlusion. In all the other cases, there was no complication near the starting point of recanalization.

## Discussion

The “catheter first” approach is somewhat contradictory to the standard approach of “wire first”. The catheter first approach uses the diagnostic catheter as the leading point throughout the entire recanalization including the re-entry in the patent distal vessel and is therefore different from the approach of using the catheter to get on purpose in the subintimal space as described by Reekers and Bolia (Reekers and Bolia 1998). Using the soft tip of a catheter seems less traumatic, leading likely to a longer intraluminal course. In all our cases, we noticed only one single technical complication requiring a longer stenting as intended because of a flow limiting dissection extending proximal to the occlusion.

Our data shows that recanalization of occluded lower extremity arteries between the aortic bifurcation and the popliteal artery can be achieved in almost half of the cases (47.8%) with the tip of the angiographic catheter only, irrespective of the localization of the occlusion. With the additional help of the guide wire (mostly soft end, less often stiffer back end), the success rate is increased by 36.9%, adding up to a success rate of 84.7%. These results are consistent with the reported success rates for crossing CTOs using a combination of hydrophilic guidewires and catheters (Reekers and Bolia 1998; London et al. 2011). When compared with the so far only published data on CTO support catheters (Cannavale et al. 2017), the first step of our approach achieves similar or slightly better technical success rates. Occlusion length was similar for the different recanalization techniques and interestingly did not influence recanalization time. On the other hand, high grade of vessel calcification significantly lowered the success rate of the catheter only approach for the entire recanalization. For heavy calcification, the stiff backend of the glidewire was most successful. Therefore, a catheter only approach seems especially useful with little or no calcification.

Interestingly, recanalization times were all strongly right-skewed when broken down according to technique. If successful, the catheter only approach was quick and each additional step was more time consuming in this step-up approach.

Applying the Pareto Principle with regard to optimization efforts, we calculated the 80th percentile of recanalization time for catheter-only recanalizations as well as recanalizations with soft- and back-end of GW, which corresponds to an 80th percentile of about 5 min., 15 min. and 30 min. Respectively. Derived from this data we suggest trying 5 min with the catheter alone, then 10 min with the soft end of the guidewire and then switching to the stiffer back-end of the guidewire for another 15 min.

Main limitations of our study are the rather small sample size, no control group and the lack of an intravascular imaging modality to differentiate between endo- and subintimal crossing.

## Conclusion

The “catheter first” approach using the catheter tip as the only leading point for the entire recanalization without a guide wire is a promising alternative method to recanalize occluded lower extremity arteries between the aortic bifurcation and the popliteal artery. The use of the catheter lead recanalization of the entire occluded length was successful in about half of the occlusions (47.8%). With the additional use of a guidewire, the technical success rose to 84.7%.

We suggest a step up approach with 5 min catheter only – 15 min catheter and soft-end glidewire – 30 min back-end glidewire.

## Data Availability

All data generated or analyzed during this study are included in this article.

## Abbreviations

CTO: Chronic total occlusion
GW: Glidewire
